# The value of plasma presepsin as a diagnostic and prognostic biomarker for febrile neutropenia in children

**DOI:** 10.1080/07853890.2025.2561224

**Published:** 2025-09-17

**Authors:** Jianbo Liang, Jinmei Luo, Gen Li, Laisheng Li, Juehui Wu

**Affiliations:** ^a^ Department of Laboratory Medicine, The First Affiliated Hospital of Sun Yat-sen University, Guangzhou, People’s Republic of China; ^b^ Department of Internal Medicine, Medical Intensive Care Unit and Division of Respiratory Diseases, The Third Affiliated Hospital of Sun Yat-sen University, Guangzhou, People’s Republic of China; ^c^ Guangdong Women and Children Hospital, Guangzhou, People’s Republic of China

**Keywords:** Presepsin, febrile neutropenia, children, diagnosis, prognosis

## Abstract

**Background:**

Febrile neutropenia (FN) is a prevalent infectious complication in paediatric cancer patients undergoing anticancer treatment. Presepsin is a reliable biomarker for sepsis; however, its clinical value in the context of paediatric FN remains unclear. This study aimed to evaluate the efficacy of presepsin in the entire course of paediatric patients with FN.

**Methods:**

In this prospective study, 108 paediatric patients with haematological tumours complicated with neutropenia were recruited, of whom 23 had febrile neutropenia. Blood samples were collected daily, and four inflammation biomarkers, presepsin, procalcitonin (PCT), interleukin-6 (IL-6) and C-reactive protein (CRP), were analyzed. Clinical and laboratory parameters were collected, and the diagnostic and prognostic value of biomarkers for FN was assessed.

**Results:**

The concentrations of presepsin, PCT, IL-6 and CRP were prospectively measured in 57 FN episodes in 23 cases of malignant haematological diseases in children. The concentrations of presepsin, PCT and CRP were significantly elevated 72 h before onset compared to those in the control group. Only the concentration of presepsin at FN onset was significantly higher than 72 h before FN onset. The area under the curve (AUC) of presepsin, PCT, IL-6 and CRP for FN diagnosis 24 h before FN onset were 0.9118, 0.8214, 0.9052 and 0.8286, respectively. For prognosis, the levels of presepsin were consistently and significantly higher at every time point in patients with unfavourable outcomes than in those with favourable outcomes.

**Conclusion:**

Presepsin demonstrated the best performance in both the early diagnosis and prognosis assessment of FN, indicating that it is a reliable biomarker for managing the entire FN process. Close monitoring of presepsin may enable earlier intervention for FN, reducing the occurrence of adverse events caused by infection.

## Introduction

Paediatric Haematology-Oncology patients receiving immunosuppressive therapy, such as chemotherapy, radiotherapy and haematopoietic stem cell transplantation, are prone to severe disease progression, serious complications and even death [1]. Febrile neutropenia (FN) is one of the most familiar complications, occurring in more than 40% of paediatric patients undergoing anticancer treatment [2]. Febrile neutropenia can delay chemotherapy regimens, diminish treatment effectiveness, prolong hospital stays and escalate medical costs. Furthermore, infections arising from febrile neutropenia can lead to sepsis, severe infections in multiple organ systems and even death [3,4]. Proper evaluation and timely broad-spectrum antibiotic treatment are essential to avoid clinical deterioration in these patients [5]. Bacterial infection is one of the main causes of febrile neutropenia, accounting for 20–30% of cases, and early diagnostic biomarkers could provide valuable evidence for physicians to initiate prompt antibiotic therapy [6]. Appropriate diagnosis may help physicians develop rational treatment, prevent unnecessary overtreatment, and reduce the health care burden. However, due to immunosuppression, clinical signs of infection may be unclear in febrile neutropenia patients, and other noninfectious causes of fever, such as malignancy itself, chemical drugs and blood transfusions, make it difficult to precisely identify the underlying factors of febrile neutropenia [7].

Several recent studies have shown that procalcitonin (PCT), serum amyloid A, C-reactive protein (CRP) and proinflammatory cytokines, such as interleukin (IL)-6, IL-8, presepsin, IL-33, vascular endothelial growth factor (VEGF), cell-free human plasma DNA (cf-DNA) and tumour necrosis factor (TNF)-α are potential biomarkers for febrile neutropenia [8–10]. Although some of these biomarkers can be helpful to support physicians in assessing infection, no international consensus based on these markers has been established as an appropriate strategy for managing febrile neutropenia [10,11]. Therefore, more appropriate biomarkers are needed to guide clinical decision-making in febrile neutropenia.

Presepsin is a soluble fragment of cluster of differentiation 14 (sCD14-ST), a co-receptor expressed on the membrane of monocytes and macrophages, and which identifies and binds numerous bacterial products, such as lipopolysaccharide (LPS) and the lipopolysaccharide-binding protein (LBP) complex [12]. Once the LPS/LBP complex binds to CD14, it triggers the activation of intracellular pro-inflammatory signalling, consequently inducing cytokine release and activation of the acquired immune response, during which a portion of the CD14 molecule is released into circulation and further degraded by proteases into the N-terminal fragment of the soluble CD14 subtype, sCD14-ST, also known as presepsin [12,13]. Although presepsin is found in healthy individuals without inflammatory disease (less than 200 pg/mL), its plasma levels are elevated in the earlier stages of bacterial infections compared to the well-established inflammation biomarkers (CRP, PCT), which are present in the blood 2 h after the initiation of infection [14–16]. Several studies have demonstrated that presepsin could be a useful biomarker for the early diagnosis, treatment and prognosis of sepsis in adult patients [17,18]. Presepsin could also serve as an accurate biomarker of sepsis in paediatric patients [19,20].

Recently, presepsin has been studied in febrile neutropenia patients and demonstrated to be a reliable biomarker for early diagnosis of infection [6,21,22]. However, most studies have been conducted in Western countries, and regional differences may affect the generalizability of these findings. In this study, we aimed to investigate the ability of presepsin to diagnose infections and determine the prognosis of febrile neutropenia patients in southern China.

## Materials and methods

### Study population

This prospective, single-centre study was conducted at the First Affiliated Hospital of Sun Yat-sen University (Guangzhou, Guangdong, China) between December 2022 and November 2023. In this study, we evaluated 183 children (aged ≤18 years) with agranulocytosis undergoing chemotherapy or haematopoietic stem cell transplantation for haematological malignancies. 44 cases were excluded due to inappropriate sampling time, incomplete marker analysis, or insufficient clinical data, and the remaining 139 patients were divided into two groups: febrile neutropenia (*n* = 23) and afebrile neutropenia (AFN) (*n* = 116) (Supplementary Figure 1). In the febrile neutropenia group, patients were further divided into two subgroups according to their outcomes during hospitalization: favourable outcomes (*n* = 18) and unfavourable outcomes (death or admission to ICU) (*n* = 5). The study complied with the provisions of the Declaration of Helsinki and was approved by the Medical Ethics Committee of the First Affiliated Hospital of Sun Yat-sen University (IRB No:2022-461). For all patients, informed consent was obtained from their guardians *via* telephone contact.

Febrile neutropenia was defined as an absolute neutrophil count (ANC) less than 0.5 × 10^9^/L or a predicted drop to this level within the subsequent 48 h, coupled with a fever of ≥38 °C. None of the patients had received antibiotics or had confirmed pathogen infections before enrolment.

### Measurement of biomarkers

Routine blood collection was performed every 1–2 days from the start of chemotherapy until the seventh day after febrile neutropenia was confirmed. The blood was centrifuged within 2 h of extraction, and the plasma or serum was stored at −80 °C until measurement.

Clinical data were collected from the hospital’s Laboratory Information System (LIS) after admission. CRP levels were measured using a Mindray BC6800 analyzer. Biomarkers, including presepsin, IL-6 and PCT, were precisely quantified using Mindray test kits and a highly automated Mindray Chemiluminescent Immunoassay Analyzer (CLIA) model CL6000i.

### Statistical analysis

SPSS software (version 26.0, IBM Corp., Armonk, NY, USA) and GraphPad Prism (version 9.0) were used for the statistical analysis. Normality of the distribution was assessed using the Kolmogorov-Smirnov test (AFN group) and Shapiro-Wilk tests (FN group). Based on the analysis results, the differences between each marker at each time point were compared using parametric or non-parametric tests. If normal distribution and homogeneity of variance were satisfied, parametric tests were used; otherwise, non-parametric tests were used. Continuous variables were expressed as median (range), and categorical variables were expressed as proportions. The diagnostic characteristics of the biomarkers were analyzed using receiver operating characteristic (ROC) curve analysis. The sensitivity (SEN) was calculated as the number of patients with positive results divided by the number of patients experiencing the outcome. Specificity (SPE) was calculated as the number of patients with negative results divided by the number of patients not experiencing the outcome. The optimal cut-off value was determined as the value corresponding to the upper left corner of the ROC curve. All statistical tests were two-sided, and statistical significance was set at *p* < 0.05.

## Results

### Demographic and patient characteristics

A total of 139 patients were enrolled and divided into two groups: FN (*n* = 23) and AFN (*n* = 116). In the FN group, we enrolled 57 febrile neutropenia episodes in 23 patients with haematological malignancies, including acute myeloid leukaemia (*n* = 5), acute lymphoblastic leukaemia (*n* = 11) and others (*n* = 7, including aplastic anaemia, Burkitt’s lymphoma, myelodysplastic syndrome, chronic myeloid leukaemia and blastic plasmacytoid dendritic cell neoplasm). Overall, the study population consisted of 16 (69.6%) male and 7 (30.4%) female patients. The median age of the patients was 7 years (range: 1–16 years). Twenty patients underwent chemotherapy, and three patients received haematopoietic stem cell transplantation (HSCT). Thirteen patients received granulocyte colony-stimulating factor (G-CSF) therapy, while the remaining 10 did not. Of the 23 febrile neutropenia patients, only 8 cases were positive in blood culture, including 3 cases of Gram-positive bacteria, 3 cases of Gram-negative bacteria and 2 cases of fungal infection. The median white blood cell count, neutrophil count and creatinine level were 1.59 × 10^9^/L (range: 0.66–7.4), 0.85 × 10^9^/L (0.01–7.02), 28.79 μmol/L (8.92–240), respectively. Of the patients, 56.5% (*n* = 13) had fever of unknown origin, 34.8% (*n* = 8) had bacteraemia and 8.7% (*n* = 2) had fever caused by septic shock. Clinical and/or microbiological data that could cause fever were also recorded. All febrile neutropenia patients received appropriate antimicrobial agents, but five cases were admitted to the ICU or died, which is considered unfavourable outcomes (Table 1). The above information about patients in the AFN group is also shown in Table 1.

**Table 1.** Patient background.

	FN	AFN
Patients, *N*	23	116
Gender, male (%)	16 (69.6%)	70 (60.3%)
Age, median (range), years	7 (1–16)	6.9 (0.08–18)
Primary disease		
Acute myeloid leukemia	5	17
Acute lymphoblastic leukemia	11	68
others	7	31
Treatment, *N*		
Chemotherapy	20	108
Allogeneic HSCT	2	5
Autologous HSCT	1	3
G-CSF use, *N*		
Yes	13	40
No	10	76
Febrile episode, *N*	57	/
Positive blood culture, *N*	8	0
G + bacteria, *N*	3	
G- bacteria, *N*	3	
Fungus, *N*	2	
Unfavourable outcome, *N* (%)	5 (21.7%)	0
White blood cell count at enrolment (10^9^/L), median (range))	1.59 (0.66–7.4)	2.79 (0.12–17.52)
Neutrophil count at enrolment (10^9^/L), median (range)	0.85 (0.01–7.02)	0.94 (0.01–5.65)
Serum creatinine at onset of febrile neutropenia (mmol/L), median (range)	28.79 (8.92–240)	33.13 (15.62–86.3)
Causes of febrile neutropenia, *N* (%)		
Septic shock	2 (8.7%)	
Bacteraemia	8 (34.8%)	/
Fever of unknown origin	13 (56.5%)	

### Assessment of presepsin, PCT, IL-6 and CRP levels

**Figure 1.**
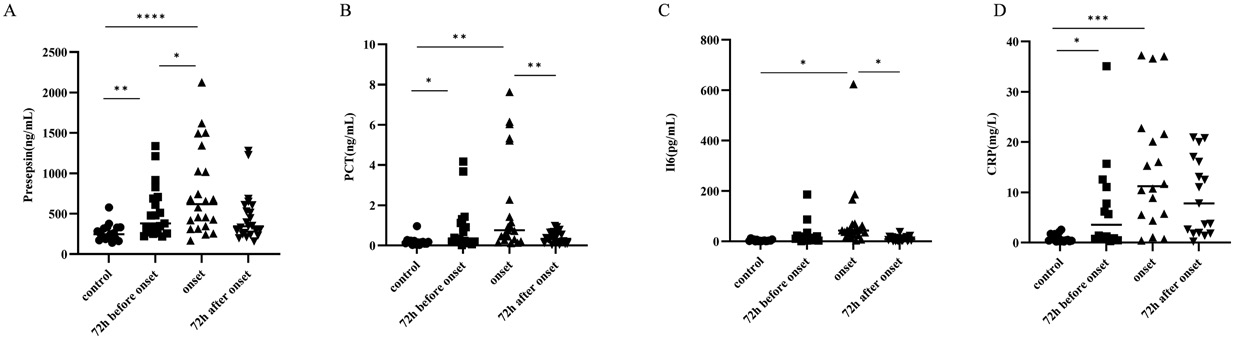
Distribution of presepsin, PCT, IL6 and CRP in different time periods. (a)The plasma level of presepsin in different time periods; (b) The serum of PCT in different time periods; (c) The serum of IL6 in different time periods; (d) The plasma level of CRP in different time periods. Control: prechemotherapy levels, onset: onset of FN levels. *p < 0.05, **p < 0.01, ***p < 0.001 and ****p < 0.0001.

Figure 1 shows the changes in the levels of each biomarker at different stages. Compared to the control, presepsin (Figure 1a), PCT (Figure 1b) and CRP (Figure 1d) levels were significantly elevated 72 h before onset (*p* < 0.05). At febrile neutropenia onset, the levels of presepsin, PCT, IL-6 (Figure 1c) and CRP were significantly higher than those in the control (*p* < 0.05). Compared with 72 h before febrile neutropenia onset, only the level of presepsin was significantly increased at the onset of febrile neutropenia.

### Early diagnostic value of presepsin, PCT, IL-6 and CRP for febrile neutropenia

**Figure 2.**
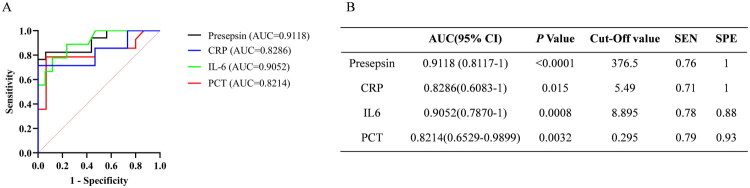
ROC curves of presepsin, PCT, IL6, CRP in 24 h before onset of febrile neutropenia. (a) ROC curves of presepsin, PCT, IL6, CRP; (b) Statistical evaluation of AUC difference of each index.

**Figure 3.**
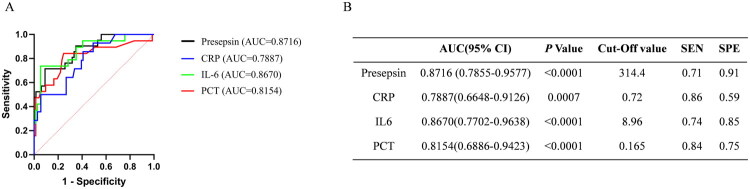
ROC curves of presepsin, PCT, IL6, CRP in 72 h before onset of febrile neutropenia in FN patients and AFN patients. (a) ROC curves of presepsin, PCT, IL6, CRP; (b) Statistical evaluation of AUC difference of each index.

We performed ROC analysis of the four biomarkers for early febrile neutropenia diagnosis at different time points. The ROC curves at 24 h before onset and control are shown in Figure 2, the AUC values of presepsin, PCT, IL-6 and CRP were calculated from ROC curves were 0.9118 (95% CI: 0.8117–1, SEN: 0.76, SPE: 1), 0.8214 (95% CI: 0.6529–0.9899, SEN: 0.79, SPE: 0.93), 0.9052 (95% CI: 0.7870–1, SEN: 0.78, SPE:0.88), 0.8286 (95% CI: 0.6083–1, SEN: 0.71, SPE: 1), respectively. The AUC values of presepsin, PCT, IL-6 and CRP at 48 h before onset and control (Supplementary Figure 2) were 0.7656 (95% CI: 0.5898–0.9414, SEN: 1, SPE: 0.44), 0.7444 (95% CI: 0.5467–0.9421, SEN: 0.58, SPE: 0.93), 0.8170 (95% CI: 0.6175–1, SEN: 0.67, SPE: 0.94) and 0.7333 (95% CI: 0.4925–0.9741, SEN: 1, SPE: 0.6), respectively. In addition, between 72 h before onset and control, the AUC of presepsin, PCT, IL-6 and CRP were 0.6912 (95% CI: 0.4293–0.9531, SEN: 0.6, SPE: 0.65), 0.5167 (95% CI: 0.1382–0.8951, SEN: 0.5, SPE: 0.73), 0.7412 (95% CI: 0.5272–0.9522, SEN: 1, SPE: 0.53) and 0.6667 (95% CI: 0.4332–0.9001, SEN: 1, SPE: 0.6), respectively (Supplementary Figure 3). Furthermore, ROC analyses of presepsin, PCT, IL-6 and CRP at 72 h before the onset of febrile neutropenia and AFN patients (Figure 3) showed that the value of presepsin (0.8716 (95% CI: 0.7855–0.9577, SEN: 0.71, SPE: 0.91)) was higher than the AUC of PCT (0.8154 (95% CI: 0.6886–0.9423, SEN: 0.84, SPE: 0.75)), IL-6 (0.8670 (95% CI: 0.7702–0.9638, SEN: 0.74, SPE: 0.85)) and CRP (0.7887 (95% CI: 0.6648–0.9126, SEN: 0.86, SPE: 0.59)). Combined with the changes of each marker before FN onset (Figure 1) and ROC analysis, it was shown that presepsin had a relatively better effect on the early diagnosis of FN.

### Prognosis value of presepsin, PCT, IL-6 and CRP for febrile neutropenia

**Figure 4.**
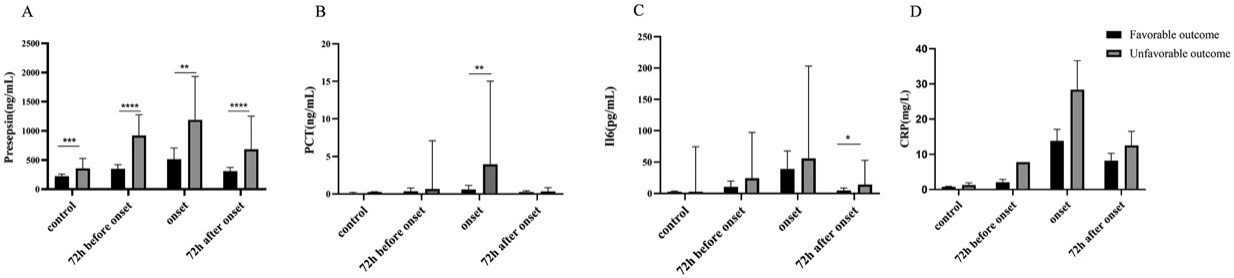
Distribution of presepsin, PCT, IL6 and CRP in FN patients with different prognoses in different time periods. (a) The plasma levels of presepsin in FN patients with different prognoses; (b) The serum levels of PCT in FN patients with different prognoses; (c) The serum levels of IL6 in FN patients with different prognoses; (d) The plasma levels of CRP in FN patients with different prognoses. Favourable outcome (*N* = 18); unfavourable outcome (die or be admitted to ICU, *N* = 5). *p < 0.05, **p < 0.01, ***p < 0.001 and ****p < 0.0001.

**Figure 5.**
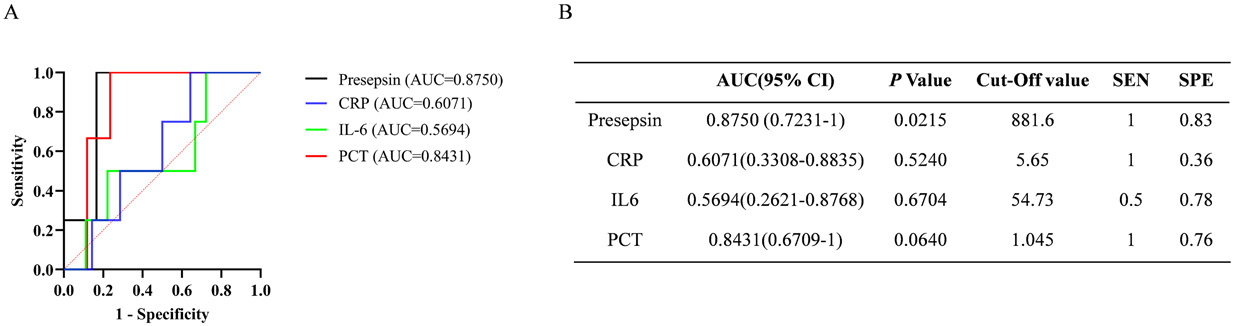
ROC curves of presepsin, PCT, IL6 and CRP in FN patients with different prognosis. (a) ROC curves of presepsin, PCT, IL6, CRP; (b) Statistical evaluation of AUC difference of each index.

According to the different prognostic outcomes, patients were divided into favourable and unfavourable outcome groups, and the differences in various biomarkers between the two groups were compared, as shown in Figure 4. Interestingly, presepsin levels were consistently and significantly higher at every time point in patients with unfavourable outcomes than in those with favourable outcomes (*p* < 0.05). PCT, IL-6 and CRP levels were also elevated to some extent at the early stage of febrile neutropenia, but the differences were not statistically significant. Meanwhile, ROC analysis showed that the AUC of presepsin (0.8750 (95% CI: 0.7231–1, SEN: 1, SPE: 0.83)) was higher than those of PCT (0.8431 (95% CI: 0.6709–1, SEN: 1, SPE: 0.76)), IL-6 (0.5694 (95% CI: 0.2621–0.8768, SEN: 0.5, SPE: 0.78)) and CRP (0.6071 (95% CI:0.3308–0.8835, SEN: 1, SPE: 0.36)) at febrile neutropenia onset (Figure 5).

Meanwhile, ROC analysis was used to evaluate the prognostic effects of presepsin, PCT, IL-6 and CRP concentrations at different time periods before febrile neutropenia onset. Supplementary Figure 4 shows that AUC values of presepsin, PCT, IL-6 and CRP in 24 h before febrile neutropenia onset were as follows: 0.8571 (95% CI: 0.0.5911–1, SEN: 0.8, SPE: 1), 0.8000 (95% CI: 0.5610–1, SEN: 1, SPE: 0.6), 0.7143 (95% CI: 0.2436–1, SEN: 0.67, SPE: 1), 0.7000 (95% CI: 0.2081–1, SEN: 1, SPE: 0.4), respectively. In addition, at 48 h before febrile neutropenia onset, the AUC of presepsin, PCT, IL-6 and CRP were 0.7407 (95% CI: 0.3711–1, SEN: 0.67, SPE: 0.89), 0.7778 (95% CI: 0.4894–1, SEN: 1, SPE: 0.56), 0.6250 (95% CI: 0.2895–0.9605, SEN: 1, SPE: 0.62), 0.6667 (95% CI: 0.1332–1, SEN: 1, SPE: 0.67), respectively (Supplementary Figure 5). The AUC values of presepsin, PCT, IL-6 and CRP at 72 h before febrile neutropenia onset were 0.6667 (95% CI: 0.1502–1, SEN: 0.5, SPE: 0.83), 0.6667 (95% CI: 0.1332–1, SEN: 1, SPE: 0.67), 0.5000 (95% CI: 0.0796–0.9204, SEN: 0.25, SPE: 1), 0.5000 (95% CI: 0.0345–1, SEN: 0.5, SPE: 1), respectively (Supplementary Figure 6). According to the above data, the change in presepsin concentration before febrile neutropenia onset also has a good predictive effect on patient prognosis.

## Discussion

Febrile neutropenia is challenged by life-threatening complications with difficult in discriminating infectious from noninfectious factors of fever [23]. In febrile neutropenia patients with immunosuppression, the indicators of infection may be insensitive or even undetectable, and other factors can affect body temperature. Therefore, a reliable biomarker is required to improve the diagnostic and prognostic value of febrile neutropenia in clinical treatment.

In this study, we comparatively assessed the diagnostic and prognostic value of presepsin in patients with febrile neutropenia. The most significant finding was that presepsin levels significantly increased from 72 h before fever onset to the febrile period, which was not observed with the other markers. The exact reason is not well known and might be explained by presepsin, being a secreted molecule that is rapidly produced by activated innate immune effector cells in response to pathogens [24]. Meanwhile, the rise in presepsin levels is significantly earlier (72 h) than the appearance of clinical symptoms (fever), we suspect that there may be several reasons: (1) According to the definition of FN, fever is defined as ≥38 °C. Through the review of positive cases, it was found that some patients had sustained low-grade fever (<38 °C) after the increase in presepsin levels before the diagnosis of FN, and the temperature exceeded 38 °C after the uncontrollable infection in the later stages. (2) Prophylactic antibiotic treatment is often administered to patients after low-grade fever in clinical practice, which may lead to further delay in clinical symptoms (fever ≥38 °C). This finding is consistent with other studies showing that plasma presepsin levels increase earlier than PCT and CRP, as a biomarker of infection [25,26]. Furthermore, we conducted ROC analysis of the early diagnostic efficacy of each marker at 72 h, 48 h and 24 h before fever onset. Presepsin showed the highest performance (AUC = 0.9118) at 24 h before fever onset, whereas IL-6 had the best early diagnostic efficacy at 48 h and 72 h before fever onset, and presepsin ranked second. Presepsin still showed the highest AUC value at 72 h before fever onset when comparing febrile neutropenia and non-FN patients, followed by IL-6, which may be related to its earliest peak levels during infection [27]. These results suggest that presepsin has excellent efficacy for the early diagnosis of febrile neutropenia, in accordance with previous studies [21,22,28].

We further evaluated the value of presepsin in assessing the prognostic outcomes of patients with febrile neutropenia. Presepsin levels were significantly higher in patients with poor outcomes than in those with good outcomes at all time points. In contrast, PCT and IL-6 levels were significantly elevated only during the febrile period and 72 h after fever, respectively, in patients with poor outcomes. ROC analysis yielded similar conclusions, indicating that presepsin demonstrated equal or even superior performance compared to other markers, which is consistent with the findings of Moustafa et al. [6]. These findings suggest that presepsin has a distinct advantage in evaluating the prognostic outcomes of febrile neutropenia patients and can effectively monitor their prognosis over time. In summary, our study objectively illustrated the dynamic changes in various markers throughout the febrile neutropenia process, confirming that presepsin is an effective marker for both the early diagnosis and prognostic assessment of febrile neutropenia.

This study had several limitations that need to be considered. First, this was a single-centre prospective investigation on a small scale, with samples drawn from the residual blood of the patients. There might have been missed cases of febrile neutropenia during the study period, leading to a limited sample size in this study, particularly in patients with poor prognoses. Second, as an observational cohort study, treatment decisions depended on the clinical physician. Third, this study did not utilize a combination of biomarkers or an artificial intelligence (AI) model to evaluate the diagnostic and prognostic value of presepsin and other biomarkers in febrile neutropenia. However, this study had several strengths. First, we excluded patients with severe liver and kidney dysfunction, ensuring that the results of various biomarkers were not confounded [29]. Second, this study encompassed the entire infection process in febrile neutropenia patients, thereby providing an objective assessment of the performance of various biomarkers at different time points during febrile neutropenia.

## Conclusions

In summary, this dynamic study of the entire process of febrile neutropenia demonstrated that presepsin exhibits superior efficacy in both the early diagnosis and prognostic evaluation of febrile neutropenia, establishing it as a reliable biomarker for the comprehensive management of this condition. Close monitoring of presepsin levels may facilitate early intervention for febrile neutropenia and reduce the incidence of infection-related adverse events.

## Supplementary Material

Supplementary Fig 6.tif

Supplementary Fig 2.tif

Supplementary Fig 5.tif

Supplementary Fig 3.tif

Supplementary Fig 1.tif

Supplementary Fig 4.tif

246578944.R2-Supplementary Fig 1.png

## Data Availability

The datasets supporting the findings of this study are available from the corresponding authors, Wu or Li, upon reasonable request. The data are not publicly accessible due to privacy or ethical restrictions, as they contain information that could compromise the confidentiality of research participants.
