# Supplementary figures and images for: The value of plasma presepsin as a diagnostic and prognostic biomarker for febrile neutropenia in children
Source: Ann Med. 2025 Sep 17;57(1):2561224. doi: 10.1080/07853890.2025.2561224 (PMC12444927; doi:10.1080/07853890.2025.2561224)

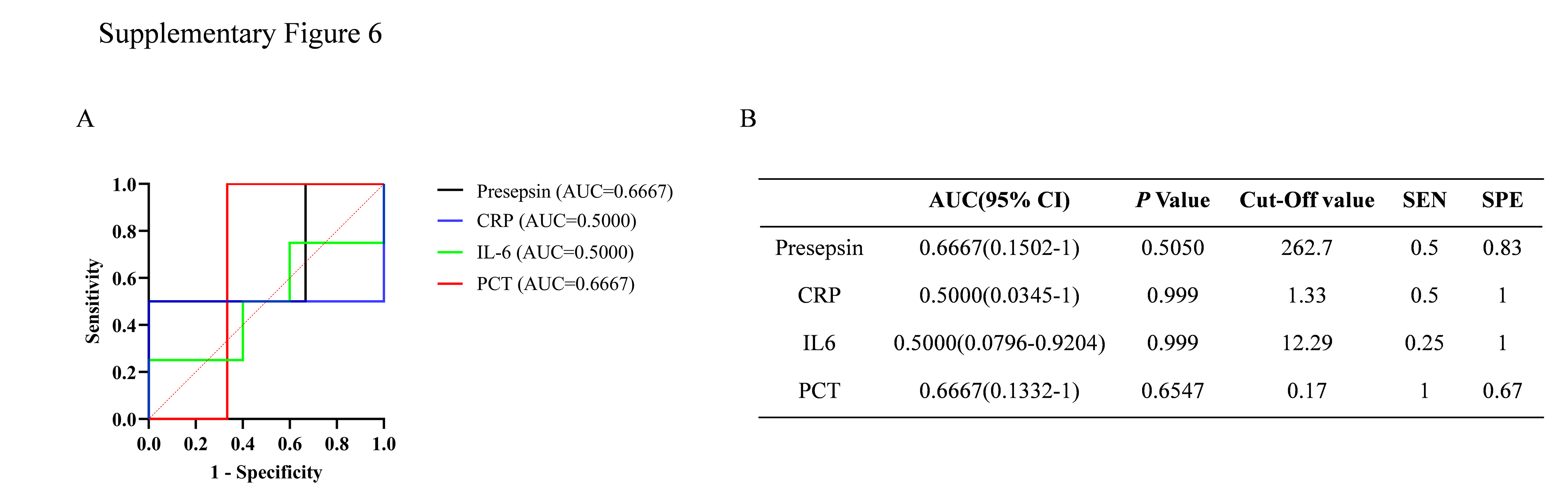

Supplement: Supplementary Fig 6.tif [file IANN_A_2561224_SM0324.tif]

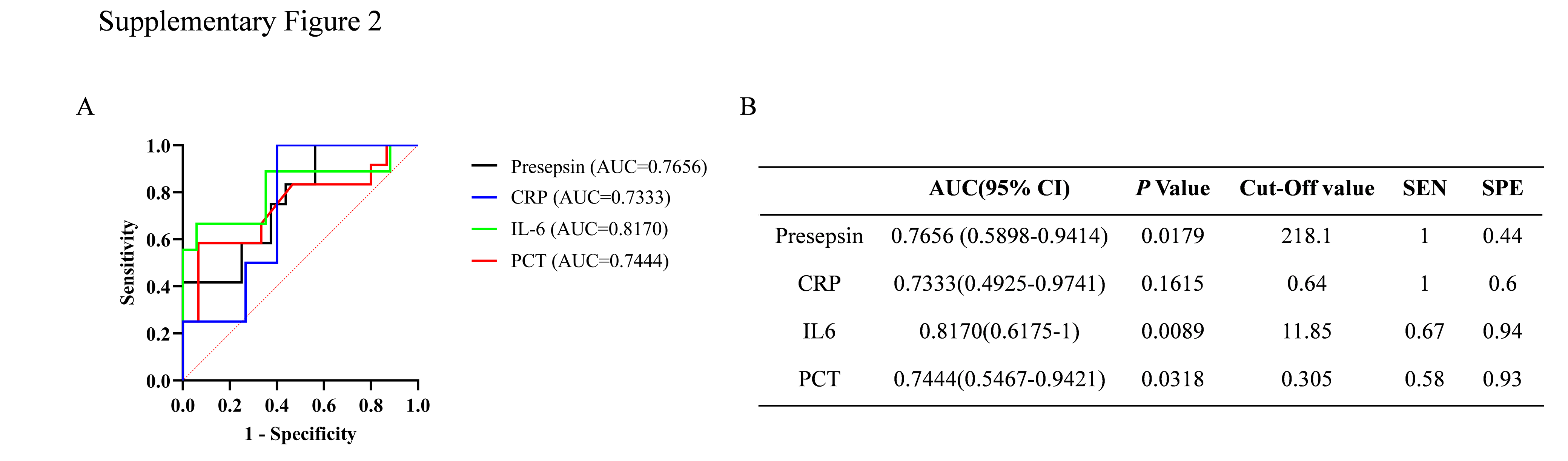

Supplement: Supplementary Fig 2.tif [file IANN_A_2561224_SM0323.tif]

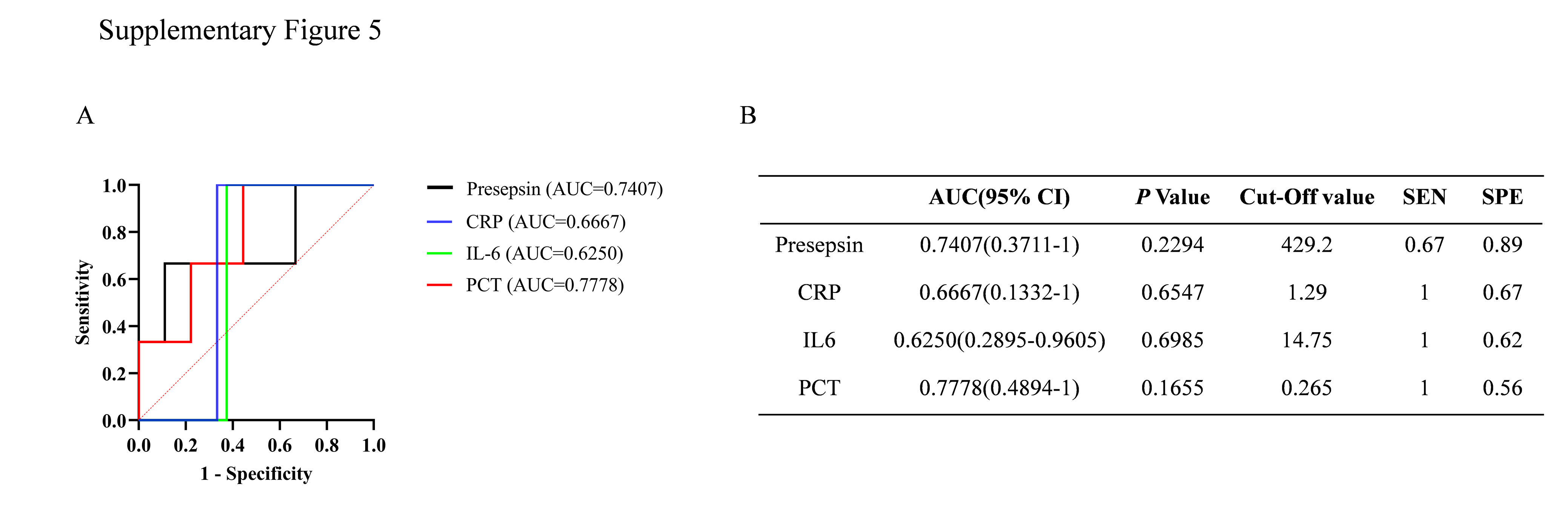

Supplement: Supplementary Fig 5.tif [file IANN_A_2561224_SM0322.tif]

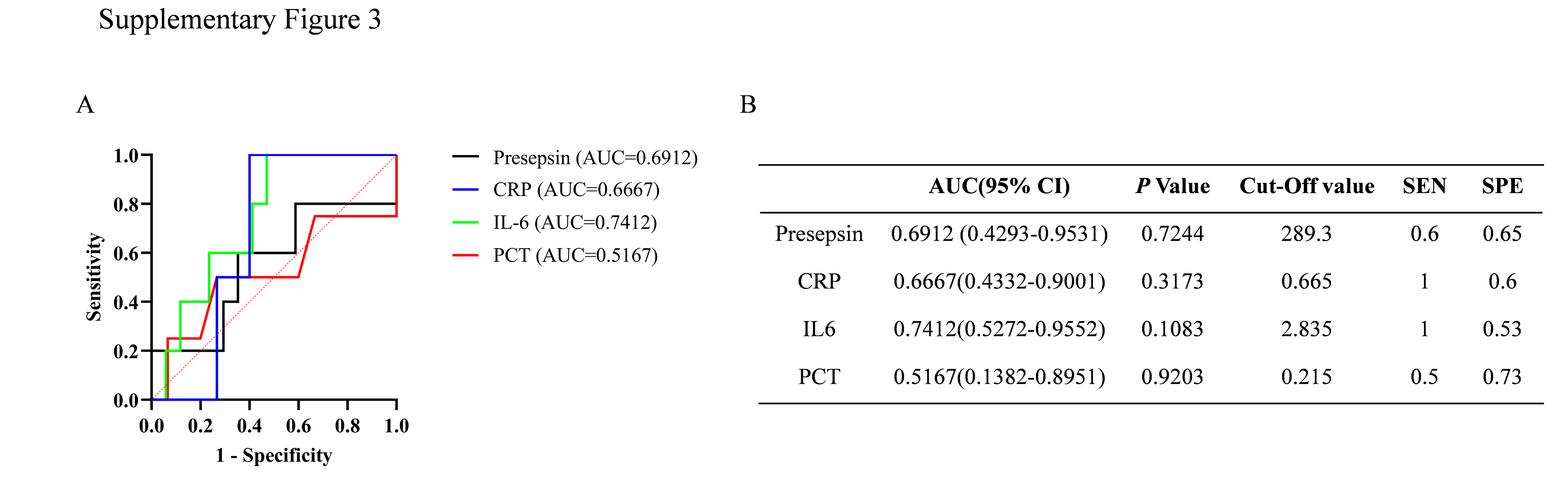

Supplement: Supplementary Fig 3.tif [file IANN_A_2561224_SM0321.tif]

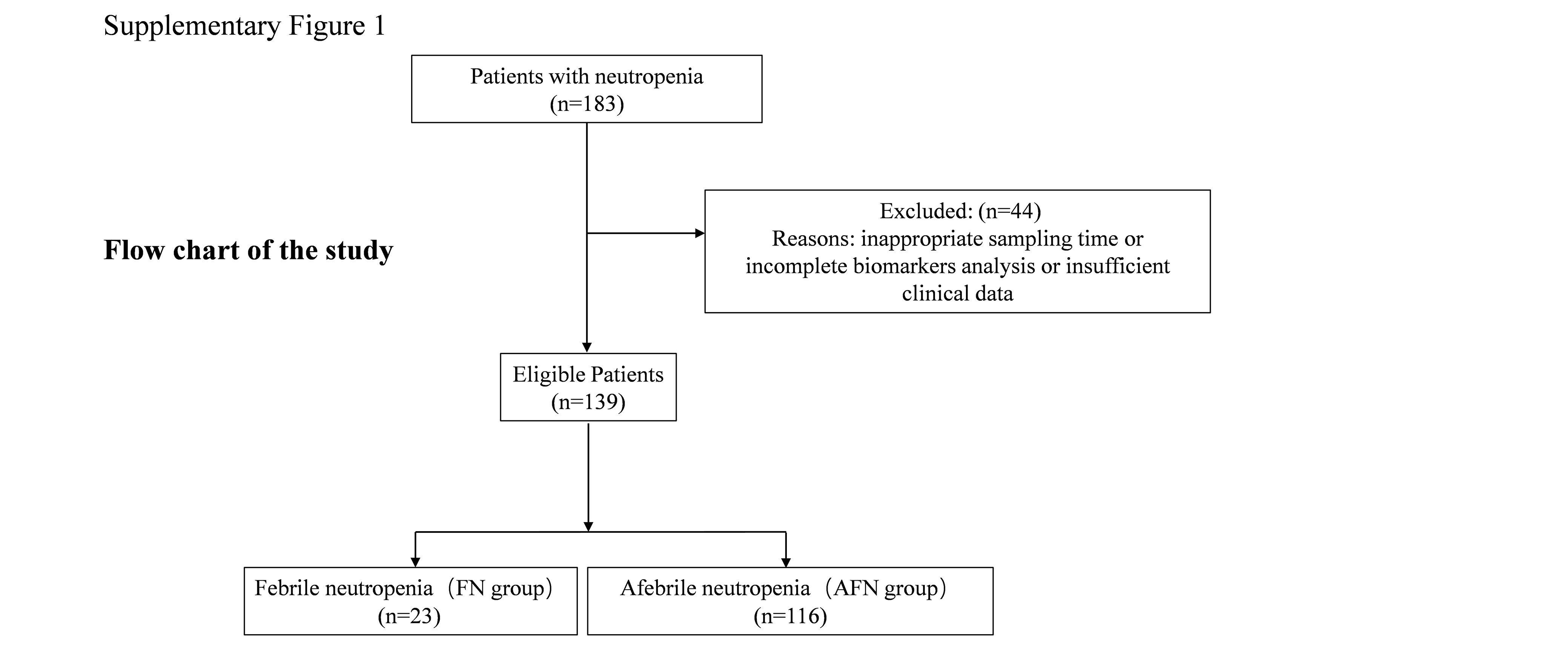

Supplement: Supplementary Fig 1.tif [file IANN_A_2561224_SM0320.tif]

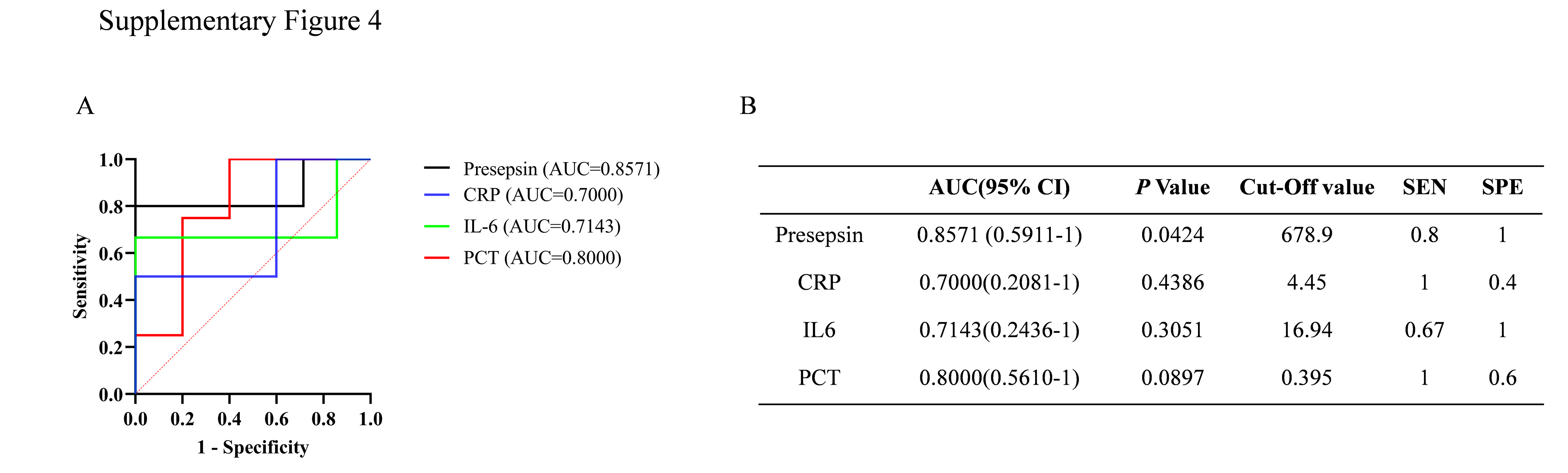

Supplement: Supplementary Fig 4.tif [file IANN_A_2561224_SM0319.tif]

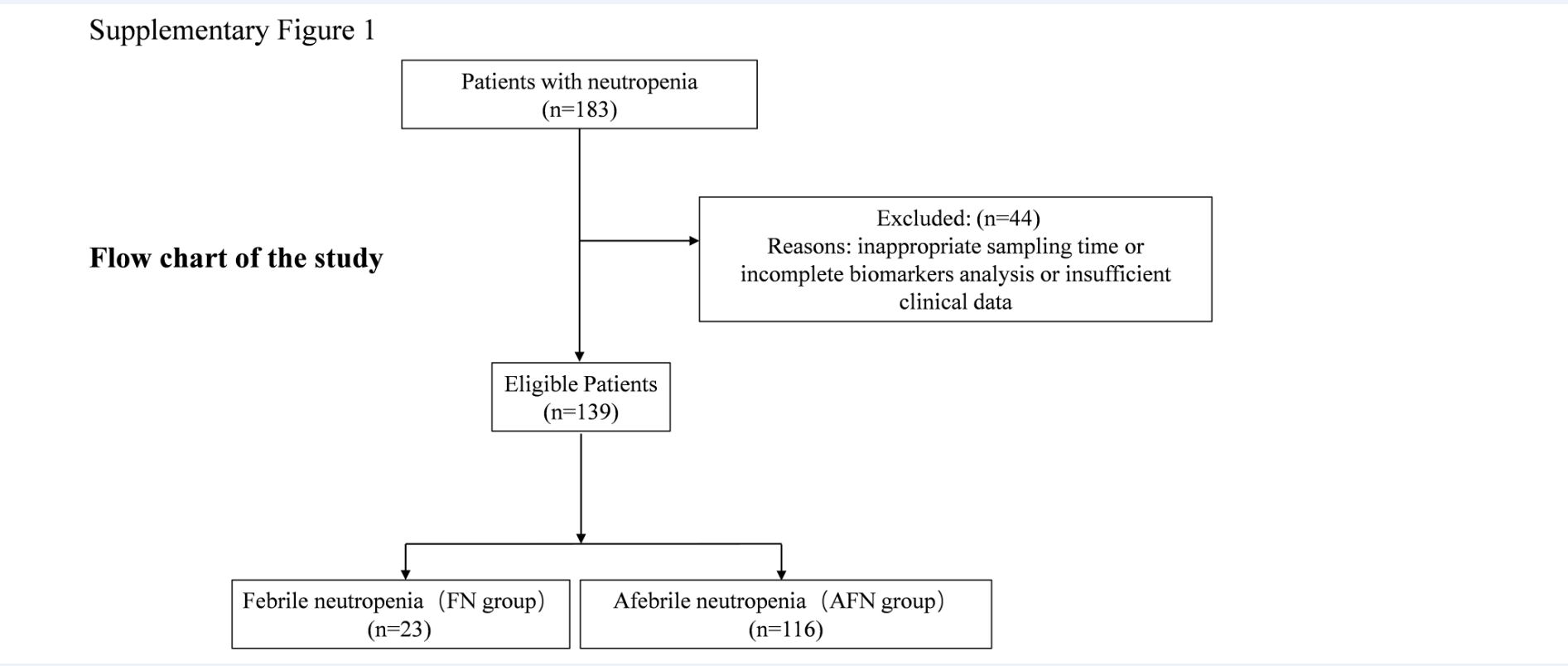

Supplement: 246578944.R2-Supplementary Fig 1.png [file IANN_A_2561224_SM0318.png]
